# The impact of aging on intestinal mucosal immune function and clinical applications

**DOI:** 10.3389/fimmu.2022.1029948

**Published:** 2022-11-28

**Authors:** Han Zheng, Chi Zhang, Qianqian Wang, Shuyan Feng, Yi Fang, Shuo Zhang

**Affiliations:** ^1^ The Second Clinical Medical College, Zhejiang Chinese Medical University, Hangzhou, China; ^2^ The First Clinical Medical College, Zhejiang Chinese Medical University, Hangzhou, China; ^3^ The Second Affiliated Hospital of Zhejiang Chinese Medical University, Hangzhou, China

**Keywords:** aging, intestinal mucosal immune, mucosal barrier, cytokine, gut microbiota

## Abstract

Immune cells and immune molecules in the intestinal mucosa participate in innate and adaptive immunity to maintain local and systematic homeostasis. With aging, intestinal mucosal immune dysfunction will promote the emergence of age-associated diseases. Although there have been a number of studies on the impact of aging on systemic immunity, relatively fewer studies have been conducted on the impact of aging on the intestinal mucosal immune system. In this review, we will briefly introduce the impact of aging on the intestinal mucosal barrier, the impact of aging on intestinal immune cells as well as immune molecules, and the process of interaction between intestinal mucosal immunity and gut microbiota during aging. After that we will discuss potential strategies to slow down intestinal aging in the elderly.

## 1 Introduction

With aging, aging cells accumulate extensively, and utilize a senescence-associated secretory phenotype to secrete a variety of extracellular regulators (1). These abnormal accumulations of aging cells will lead to potentially deleterious effects, especially a dysfunctional immune system. The dysfunctional immune system in the elderly is associated with two processes – the decline of immune function, commonly termed the “ immunosenescence” process (2), and the dysregulation and hyperactivation of inflammation, leading to a persistent chronic low-grade inflammatory state in aging individuals, commonly termed the “inflammatory aging “ process (3). As the largest immune compartment, intestinal mucosal immune function is strongly influenced by aging, which will promote the emergence of diseases, including intestinal infections, intestinal tumors, malnutrition, chronic constipation, and other age-related diseases (4).

A critical function of the intestinal mucosa is to form a barrier that separates harmful antigens of the luminal content from the body. A monolayer of polarized intestinal epithelial cells (IECs) plays a physical isolation role and constitutes the last line of defense. Meanwhile, an apical junctional complex confers the property of selective permeability to the intestine (5). Apart from the last line of defense, the extracellular components of the barrier play an important role in maintaining the integrity of the intestine (6). For example, mucus, secreted by specialized epithelial cells, prevents large particles from directly contacting with IECs (5). Antimicrobial peptides (AMPs) have broad-spectrum and high-efficiency bactericidal activity (7). Additionally, gut microbiota influences the balance between pro-inflammatory and regulatory responses. Certain members of the microbiota colonizing the intestine stimulate the production of microbicidal peptides and secretory immunoglobulin A (sIgA) and are endowed with anti-inflammatory properties. Others promote effector immune responses and are endowed with pro-inflammatory properties (8).

The gut-associated lymphoid tissue along with immune molecules are the major participants of both innate and adaptive immune responses. The gut-associated lymphoid tissue mainly includes immune cells, either interspersed between epithelial cells or present in the lamina propria, scattered or grouped in lymphoid follicles, either isolated or aggregated to form Peyers’patches in the ileum (9). Intestinal innate immune system includes intrinsic lymphocytes, macrophages, dendritic cells (DC), eosinophils, IEC, etc. (10). Cells from the immune system spectrum along with IEC are capable of using pattern recognition receptors(PRR) to recognize highly conserved pathogen-associated molecular patterns. Compared with adaptive immune system, it provides an acute nonspecific immune defense. Moreover, the PRR family links the innate and adaptive immune system by producing cytokines and costimulatory molecules (11). By contrast, the intestinal adaptive immune system utilizes highly mutated receptors that recognize specific microbial antigens for the activation and differentiation of T cells. Then, naive T cells can differentiate into either effector T cells to fight pathogens and toxins or regulatory T (Treg) cells to tolerate presence of specific antigens. In addition, B cells and their antibodies (mainly IgA) constitute the other half of adaptive immune response. B cells produce high-affinity, monoreactive antibodies primarily against pathogens and toxins through a T cell-dependent pathway. By contrary, they produce low-affinity, polyreactive antibodies primarily against commensal microbiota through a T cell-independent mechanism (12).

Aging impairs the intestinal mucosal barrier, which brings about the intestinal mucosal immune dysfunction. Also, aging adversely affects the function of intestinal mucosal immune system – protective immune response and induction of immune tolerance. Moreover, the gut microbiota in the elderly is closely associated with the aging intestinal mucosal immune system. Understanding the age-related process of intestinal mucosal immune dysfunction may help to slow down intestinal aging in the elderly, and further help to reduce the risk of age-associated diseases.

## 2 The impact of aging on the intestinal mucosal barrier

### 2.1 The impact of aging on intestinal stem cells

Intestinal stem cells (ISCs) located at the base of the intestinal crypt differentiate to produce all cell lineages of the intestinal epithelium, including enterocytes, goblet cells, Paneth cells, enteroendocrine cells, and Tuft cells. ISCs complete the renewal of the intestinal epithelium and are essential for maintaining the intestinal mucosal barrier (13).

Aging affects the external structure of crypt and the number of total cells in crypt. In small intestine of aging mice, researchers observed a decrease in the number of crypts, an increase in crypt length and width, an increase in the number of cells in per crypt and an increase in villus length (14). In colon of aging human, researchers observed increased percentage of Ki67 positive cells and decreased number of cell divisions (15). Ki67 is a widely-used proliferation marker, whose effect on cell proliferation varies by cell type (16). This suggests that increased Ki67 index in aging crypt is associated with decreased cell divisions. In addition, further study found that the expression of the cell cycle regulator CDKN1C in aging crypt is reduced, at the same time, terminal deoxynucleotidyl transferase dUTP nick end labeling (TUNEL) positive cells are increased (14). Consequently, the mechanism for the reduced cell proliferation may be associated with the reduced cell division and the reduced survival of aging cells. Cell proliferation within crypts is the principal driving force for cell migration along the villus (17). So the impact of aging on the mitotic process of crypt cells is likely to reduce the rate of IEC migration along the villi.

Aging affects the capacity of ISCs in crypt. Researchers have assessed the number of ISCs during aging in different subjects using different ISC markers and derived different results. Researchers studied on aging human using the well-established ISC marker – Olfm4, and found an increase in the number of ISCs (15). A study on Drosophila found similar results – aging ISCs are activated in response to tissue damage or infection and then become highly proliferative (18). However, researchers studied on aging mice using a variety of markers specific to ISC, including Lgr5 and Olfm4, and found no significant change (14). Overall, these results at least suggest that ISC number do not decrease with aging. Thus, although the nature of mitotic cells in the crypts has not been clarified currently, the above results suggest that the cells with reduced mitosis in the crypts are not dominated by ISC.

**Figure 1:**
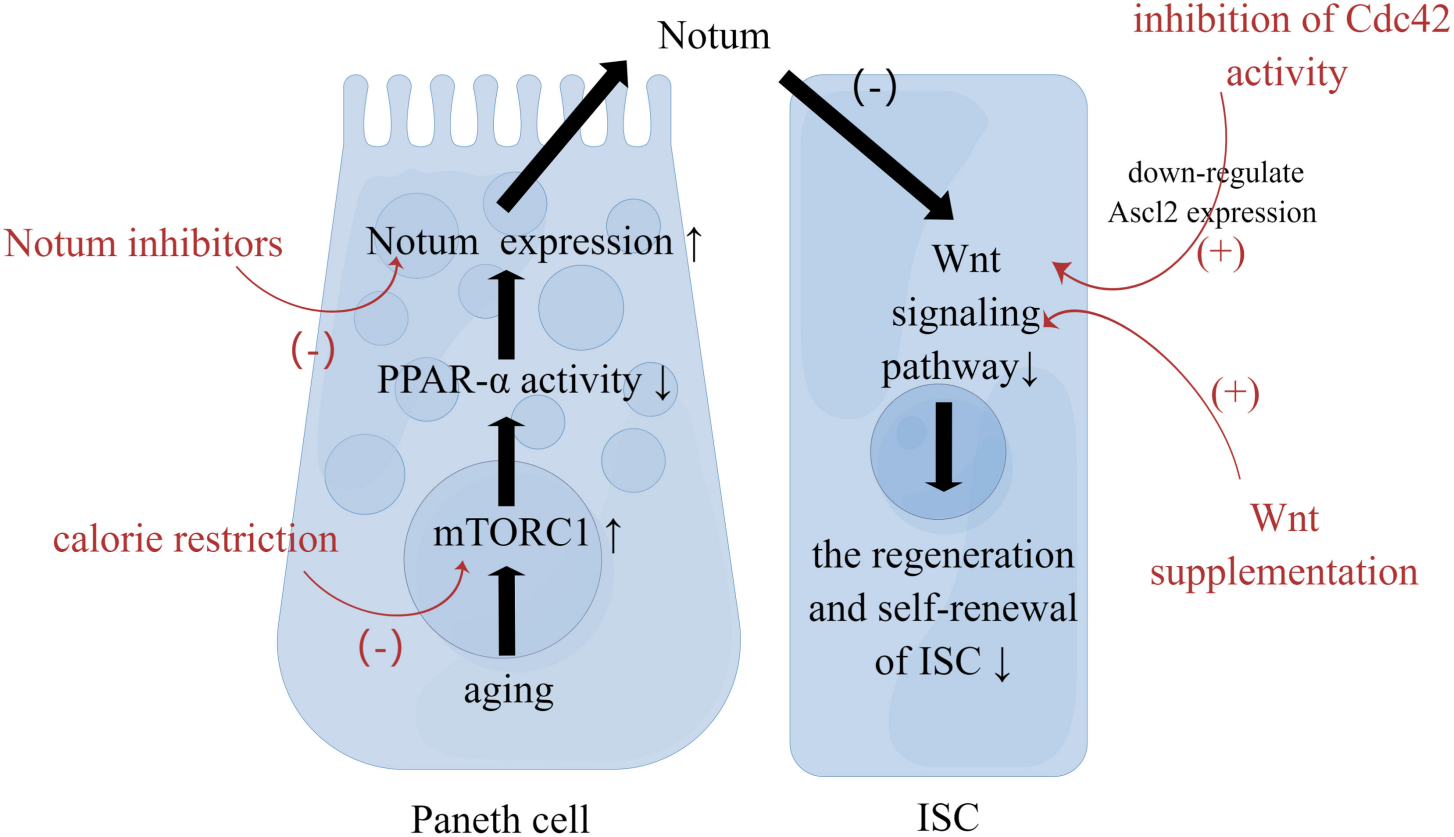
Strategies to Restore the Function of ISC. Paneth cells reside in proximity to ISCs. With aging, increased mTORC1 activity in Paneth cells alters the expression of Notum. Then Notum can diminish Wnt activity in ISC to impair function of aging IEC. Calorie restriction, Wnt supplementation, Notum inhibitors, and inhibition of Cdc42 activity can restore ISC function through the mechanism mentioned above.

Aging affects signaling within the ISCs. First, the Wnt pathway is closely associated with stem cell maintenance and differentiation in the intestinal epithelium. Aging impairs the regenerative and self-renewal capacity of ISCs, which is closely associated with a decline in the canonical Wnt signaling (19, 20). On the one hand, with aging, the expression of canonical Wnt is reduced, including the expression of Wnt3 in Paneth cells, mesenchymal cells and ISCs. On the other hand, with aging, the expression of target genes of canonical Wnt signaling and genes regulating ISC function, including β-catenin, Ascl2, and Lgr5, in ISCs or crypt is decreased as well (21). The mechanism of the decreased Wnt signaling is associated with aging Paneth cells. Increased mTORC1 activity in aging Paneth cells alters the expression of Notum through the mTORC1-PPAR-α axis (**Figure 1**). Notum is a secreted Wnt deacylase that disengages Wnt ligands from receptors on ISCs and reduces Wnt activity during development. Hence the increased expression of Notum can impair function of aging IECs (22). Moreover, the reduced function of ISC may be related to the activity of Cdc42. The increased activity of Cdc42 in aging crypt and ISC is associated with the reduced expression of Ascl2(achaete scute like 2) (23). Ascl2 is a β-catenin-dependent transcription factor that controls ISC function, so the reduced expression of Ascl2 is closely associated with the aging of ISCs (14). Second, three major signaling pathways, Wnt, Notch, and MAPK, determine the spectrum of ISC differentiation (24). Several reports have suggested changes in the composition of IECs during aging, which will be analyzed in detail below. In addition, the stress response signaling pathway to tissue injury or infection is involved in the heterotypic proliferation of ISC. During aging, ISCs exhibit up-regulated JNK signaling as well as up-regulated platelet-derived growth factor (PDGF)/vascular endothelial growth factor (VEGF) signaling, and normal Nrf2 signaling is interrupted (25, 26). As a result, ISCs are hyperactivated, highly proliferative and eventually heterogeneous. This seems to explain the increased incidence of intestinal tumors in the elderly.

### 2.2 The impact of aging on IECs

Several reports showed that the composition of IEC changed with aging for the reason that aging affects the differentiation of ISCs. Of note, many investigators have reported increased number of secretory cell lineages in aging individuals, including Paneth cells, goblet cells, enteroendocrine cells (14, 27, 28). Some of them speculated that it may be related to persistent damage to the epithelium (28), while others suggested that this alteration may be driven by changes in spectrum of ISC differentiation due to signaling pathways (14). Notch pathway is one of possible signaling pathways. Notch signaling affects ISC differentiation by regulating helix-loop-helix transcription factor Atonal homolog 1 (ATOH1). It was found that Notch1, one of the Notch receptors, and the Atoh1 gene exhibit reduced expression in aging ISCs. ATOH1 can regulate lateral inhibition through Delta-like notch ligand and secretory lineage genes (14). Paradoxically, distinct age-related changes in the secretory cell lineage have also been reported by investigators. Investigators have successively observed a decreased number of Paneth cells in aging mice and humans (29, 30). It was also found that no significant changes in enteroendocrine cells with aging (31). As for goblet cells, a decrease in the number of goblet cells has been observed at the end of the ileum and in the colon (31–33). These changes may be due to the decreased function of ISCs and increased apoptosis of IECs. Overall, more experimental data are needed to clarify the impact of aging on IEC composition, especially in different regions.

The entry of antigens from the intestine into host tissue is restricted by the IECs. M cells, as the specialized epithelial cells, can sample luminal antigens across the IECs (34). M cells develop from ISCs and their differentiation requires NF-κB receptor activator ligand/NF-κB receptor activator signaling pathway activation, or RANKL/RANK signaling pathway activation as it is often called. Also, their differentiation requires chemokine CCL20-mediated chemical attraction (35). Their maturation is mediated by the expression of E26-transformation-specific-family transcription factor Spi-B. And, glycoprotein 2 (GP2), as one of the target genes of Spi-B, has an extremely important role in the maturation of M cells (36, 37). Aging affects the differentiation and maturation of M cells. First, the overall size of follicle-associated epithelium, density of M cells in follicle-associated epithelium, expression of CCL20, and M cell-specific expressed genes are significantly reduced in Peyer’s patches. Additionally, the expression of RANKL and RANK as well as their signaling are not altered. These results suggest that the mechanisms of the impact of aging on M cell differentiation are related to actors downstream of RANKL-RANK signaling and changes in the chemokine CCL20. Second, the number of Spi-B^+^ cells, Spi-B expression, and the number of GP2^+^ cells are also significantly reduced (38, 39), suggesting that aging affects M cell maturation due to the changes in the expression of Spi-B and its target genes. Moreover, the density of mature M cells in follicle-associated epithelium of Spi-B-deficient mice and CCR6-deficient mice is significantly reduced (40, 41), resulting in reduced antigen-specific mucosal immune responses (42). It supports the above view further. In conclusion, aging adversely affects the differentiation and maturation of M cells, hindering the process of sampling and uptaking luminal antigens.

Aging affects the function of Paneth cells. On the one hand, Paneth cells are the main source of AMPs, which include defensin peptides, lysozyme C, phospholipases, C-type lectins (43). Aging affects the production of AMPs. It was shown that the expression of α-defensin (α-defensin) and lysozyme in aging mouse Paneth cells is significantly reduced, suggesting that Paneth cell dysfunction affects AMP expression. Paradoxically, this experiment found that the expression of C-type lectins Reg3b and Reg3g, β-defensin 1, and Relmb, was significantly increased at the same time. The researchers reckoned that the change of the latter may be related to age-associated inflammation and persistent damage to the aging intestinal epithelium (28). On the other hand, Paneth cells produce multiple signaling factors, including EGF, TGF-a, Wnt3, Delta-like ligands and cyclic ADP ribose (cADPR), to maintain ISC capacity and regulate ISC function (44). It was found that with aging, Paneth cells could not support the function of young ISCs as well as before. Moreover, co-culture of aging ISCs with young Paneth cells could partially recover from age-related ISC dysfunction (22). This suggests that, in addition to Notum expression, aging Paneth cells can also regulate ISC capacity and function through some signaling factors. Notably, researchers observed no change in the expression of Wnt3 or EGF in aging Paneth cells, while the expression of cADPR was reduced (22). More experiments are needed to clarify the effects of other signaling factors.

### 2.3 The impact of aging on apical junctional complex

The apical junctional complex includes tight junctions (TJs) and adherens junctions (21). TJ proteins are multiprotein complexes composed of transmembrane proteins (including claudins, ocludins, TAMPs, and JAMs), scaffolding proteins (including ZO-1/2/3 and cingulin), and regulatory molecules (45). The TJ is the principal determinant of mucosal permeability. The primary function of TJs is to strictly regulate the passage of ions through small pores that show size and charge selectivity, and macromolecules through leak pathways that do not show charge selectivity (5). Recently, numerous researchers have assessed changes in intestinal physical barrier on human, nonhuman primates, and rodents during aging. They obtained roughly the same results – the permeability of aging intestine increases, which is associated with changes in the expression of key TJ proteins (21, 34, 35, 46). Nevertheless, there are slight differences in details in these researches above. One is that there are different results about the impact on macromolecular permeability: the intestinal permeability of aging rodents and non-human primates to macromolecules increases, while aging human’s increased intestinal permeability is limited to solutes rather. In other words, aging human’s intestinal permeability to macromolecular is not affected by aging. It suggests that human’s increased intestinal permeability is not due to disturbances to the overall morphology of the TJ. The other is that the key TJ proteins affected by aging of different species are completely different: reduced expression of JAM, ZO-1 and ocludin is observed in the intestine of aging rodents and non-human primates, while no changes about the expression of the above proteins is observed in the intestine of aging human. Only increased expression of claudin-2 is observed in human. Claudin protein, as one type of TJ proteins, can be divided into sealing proteins that reduce permeability and pore-forming proteins that increase permeability. Claudin-2 belongs to the latter (36), so the increased expression of claudin-2 in the aging human intestine can increase the intestinal permeability to pathogens and toxins, which is associated with the chronic low-grade inflammatory state and higher risk of intestinal infection in the elderly. In conclusion, the intestinal permeability of human is less affected by aging than other animals. It seems that the overall morphology of the TJ suffers less for the reason that TJ protein expression changes less.

### 2.4 The impact of aging on extracellular components

As mentioned above, aging affects the production of AMP. In addition, aging affects the thickness and function of mucus. Goblet cells are the main source of mucus, which includes mucins (major components), immune mediators that regulate the intestinal microbiome, and other molecules with unknown function (37). Aging affects the thickness and function of mucus. First, aging affects the thickness of the mucus layer. In aging mice, the thickness of the mucus layer in colon is reduced (32, 33, 47). Supplementation of different gut microbiota may prevent or exacerbate such changes in aging mice, suggesting that aging may affect mucus through the gut microbiota (47). In aging human, the mucus layer thickness of stomach and duodenum in H. pylori-positive individuals is reduced, yet aging does not seem to affect H. pylori-negative individuals’ (48). The intestinal mucus plays an important role in protecting the epithelial surface from pathogens. Overall, these changes may imply a loss of mucus protection. Second, aging affects the adhesion of mucus to the gut microbiota. The adhesion of mucus to the gut microbiota, considered as a precondition for the initial colonization and subsequent proliferation of the gut microbiome, is associated with the glycan-rich structural domain of mucins (30). Unfortunately, some researchers found that the mucosal adhesion to Bifidobacterium decreases with aging in human (49, 50), which well explains the reduced abundance of Bifidobacterium in the gut microbiota of the elderly. The exact mechanism is not clear. It may be due to the changes in chemical structure of mucin, but there is no evidence that the chemical structure of mucin is impaired during aging. Bifidobacterium, as probiotics in the intestine, play an important role in intestinal homeostasis and health (51). Therefore, the reduced abundance of Bifidobacterium in the gut microbiome of the elderly is universally considered as an undesirable change that adversely affects intestinal mucosal immune function.

## 3 The impact of aging on the intestinal immune system

### 3.1 The impact of aging on immune cells

#### 3.1.1 The impact of aging on DC

DCs induce a balance of regulatory and effector T cell responses and play a critical role in intestinal immune regulation. The DC-induced balance of T cells is finely regulated by immune mediators (52). At steady state, intestinal DCs can induce regulatory T cells (Treg) to tolerate presence of soluble antigens and commensal microbiota (53). TGF-β, IL-10, Retinoic Acid and other immune mediators are required for the differentiation process to Treg (54). For example, the exposure of Retinoic Acid can lead to the upregulation of CD141 and GARP on DCs, to enhance the ability for inducing Treg. It was found that aging DCs in human show an impaired response to Retinoic Acid, resulting in reduced upregulation of CD141 and GARP on DCs and defects in the induction of Treg (55). In a word, aging affects the process of introducing Treg for tolerance of antigens and increases the susceptibility to diseases ultimately. However, upon pathogen stimulation, antigen-presenting cells recognize antigens through pattern recognition receptors and transfer them to gut-associated lymphoid tissue. Then, intestinal pathogens and their metabolites can provide immunostimulatory signals to DCs (56). Hence, previous balance is altered and DCs induce a series of effector T cells, providing antigen-specific protective immune responses against pathogens and toxins (52). During this process, CTLA-4 expression in DCs regulates CD80/86 costimulatory molecules to activate immune responses (57). In aging mouse, DCs produce less IL-12p70 and IL-15 and express less CD80/CD86 costimulatory signals, resulting in reduced antigen-specific T-cell responses (58). In contrast, providing DCs from young mice to T cells originally from older animals can introduce optimal expression of CD80 and CD86 costimulatory molecules, restoring their original ability to introduce antigen-specific T-cell responses (59). This also proves that aging affects the process of inducing effector T cells for antigen-specific protective immune responses, and enhances inflammation ultimately.

#### 3.1.2 The impact of aging on T cells

Aging diminishes the immune function of T cells, which adversely affects mucosal immune function (60). First, aging affects the proliferative response of CD4^+^ T cells in Peyer’s patches. The underlying mechanisms may be that aging altered CD4^+^ T cell responsiveness to T cell growth factors and decreased cytokine synthesis by CD4^+^ T cells (61). For example, IL-4, as one of B cell stimulation and growth factors, is secreted less by CD4^+^ T cells in aging nasal mucosa (62). Second, aging affects the expression of immunomodulatory molecules in LP CD4^+^ T cells. PD-1 and CTLA-4, as the inhibitory moleculules, show reduced expression. Also, the expression of Ki67 is decreased, which is associated with steady-state proliferation and renewal of cells (63, 64). The inhibitory molecules CTLA-4 and PD-1 can inhibit T cell activation and T cell responses through different mechanisms (65, 66). Therefore, age-associated low CTLA-4 and PD-1 expression in LP CD4^+^ T cells may be associated with CD4^+^ T cell hyperactivation as well as mucosal inflammatory environment in aging individuals. However, Ki67 is a widely-used proliferation marker, whose effect on cell proliferation varies by cell type (16). Also, Ki67 expression is generally low in LP CD4^+^ T cells from young individuals as well. So, it is difficult to determine the significance of further reduction of Ki67 in aging LP CD4^+^ T cells (63, 64). In addition, aging reduces the frequency and function of LP CD4 Th17 cell, which is important to antigen-specific immune responses and mucosal barrier integrity. LP CD4 Th1 and Th17 cells produce IFN-γ (produced mainly by Th1) and IL-17, which are critical cytokines driving immune responses against pathogenic microorganisms (67). Also LP CD4 Th1 and Th17 cells produce IL-22 (produced mainly by Th22 but also by Th1 and Th17 cells) which acts on epithelial cells to maintain the integrity of the epithelial barrier (68). It was found that the capability of Th17 and Th1 cells from the elderly is defective, including responding to gut microbiota and the proliferation after exposure to antigens (63). Considering that the absence of intestinal Th17 cells is associated with epithelial barrier damage and microbial translocation (69, 70), it is likely that defects in Th17 and Th1 cells in the elderly play an important role in the aging of the gut mucosal immune system.

### 3.2 The impact of aging on cytokine production

During aging, cytokine expression patterns are remodeled and gradually tend to a pro-inflammatory phenotype, which is referred to as “inflammatory aging” (67).

Several studies have evaluated the secretion of anti-inflammatory and pro-inflammatory cytokines in the circulation during aging. Several evidences suggested that the age-related elevations in the circulation of the pro-inflammatory cytokines IL-1, IL-2, IL-6, IL-18, and TNF-α as well as anti-inflammatory TGF-β, which are associated with various age-related diseases (67). The dysregulation of pro-inflammatory and anti-inflammatory cytokines in the circulation is undoubtedly directly related to systemic inflammation and systemic immunity. Moreover, recent studies have suggested that this dysregulation is also closely linked to intestinal mucosal immune function. Fist, the age-related elevations in the circulation of TNF, IL-2, IL-6 and IL-8 directly affect the gut microbiome in aging individuals. The link between cytokines and gut microbiome may be related to potential pathways associated with amino acid (68). In addition, the age-related elevations in the circulation of IL-6, TNF-α, IL-1β and IL-12 appear to be associated with increased intestinal permeability (69).

Currently, many studies on mice, rats, and baboons have evaluated the secretion of cytokines in the intestinal mucosa during aging. These studies have found that the secretion of pro-inflammatory cytokines IL-6, TNF-α, IFN-γ, IL-1β increases, the secretion of anti-inflammatory cytokines TGF-β, IL-4 decreases, and the secretion of IL-10 shows opposite results in rats and mice (58, 69, 70). In the intestinal mucosal immune system, cytokines are involved in the regulation of intestinal mucosal immune function. First, aging affect production of cytokines from IECs, some of which can remodel TJ proteins. It impairs the integrity of TJs, which could be a potential mechanism for increased permeability. For example, IL-1β can increase TJ permeability through activation of the standard NF-kB pathway, activation of MLCK gene and post-transcriptional degradation of ocludin mRNA (71). And, IL-6 can increase TJ permeability by upregulating claudin-2 expression. The underlying mechanism may be that IL-6 induces claudin-2 gene expression through activating the JNK signaling pathway to upregulate the expression of transcription factor AP-1 or through the MEK/ERK and PI3K pathways to upregulate the expression of transcription factor Cdx2 (72, 73). Also, TNF-α can increase TJ permeability by upregulating claudin-2 expression and downregulating occludin expression. The underlying mechanism may be that TNF-α upregulates MLCK expression, promotes MLC phosphorylation levels, activates hypoxia-inducible factor-1α, NF-κB as well as PI3K/Akt (74), and diminishes occludin promoter activity (75). In addition, IFN-γ can increase TJ permeability by upregulating claudin-2 expression and downregulating occludin expression, which may be associated with serine protease (76). So, serine protease inhibitor could be a potential strategy to slow down intestinal aging. And IFN-γ can decrease ocludin promoter activity as well (77). Second, age-related cytokines affect the regulation of epithelial cell dynamics. For example, TNF-α can inhibit cell proliferation, inhibit cell migration and induce apoptosis to interfere with the inherent recovery potential of IECs (78). Also TNF-α can synergize with IFN-γ to induce caspase-8-JAK1/2-STAT1-dependent death of IECs (79). Finally, age-related cytokines influence the regulation of the immune response. Cytokines, as the key modulators of immunity, participate in nearly all aspects of immunity. Cells from the immune system spectrum and IEC secrete pro-inflammatory cytokines linking innate and adaptive immune responses. And thereafter, these cytokines play a decisive role in the activation, differentiation, maturation and function of the immune cells (**Table 1**).

**Table 1:** Age-related cytokines affect the regulation of the immune response.

Cytokine	Age- Related Change	Major Function	Clinical Application in intestinal diseases	Ref
IL-6	increase	IL-6 orchestrates lymphocytes and stimulates B cell to secrete IgA.	Therapeutic strategies for the blockade of IL-6 are used for Inflammatory bowel disease (IBD).	(80, 81)
TNF-α	increase	TNF-α regulates interactions between IECs and immune cells.	Therapeutic strategies for the blockade of TNF-α are used for IBD.	(82, 83)
IFN-λ	increase	IFN-λ orchestrates innate and adaptive responses to participate in antiviral, antifungal and antiprotozoal defense.	Therapeutic strategies for the blockade of IFN—λ are chiefly used for systemic disease at present. Some studies suggest that the blockade of IFN—λ may alleviate intestinal inflammation.	(84–86)
TGF-β	decrease	TGF-β promotes Treg differentiation to suppress T cell proliferation and activation, mediates IgA class-switch to promote IgA production, and controls the formation as well as maintenance of gut-resident memory T Cells.	TGF-β signal restoration is a potential therapeutic strategy for IBD, such as Smad7 antisense oligonucleotide-based therapy.	(87–89)
IL-4	increase	IL-4 participates in antiparasite defense, and suppress Treg differentiation.	No therapy has been approved for intestinal diseases to date.	(90, 91)
IL-10	Not yet known	IL-10 acts on leukocytes for immune suppressive functions	Recombinant IL-10 has no effect on Crohn’s Disease, and no therapy is approved for other intestinal diseases.	(92, 93)

### 3.3 The impact of aging on immunoglobulins

On the mucosal surface, secretory immunoglobulin M (sIgM) and sIgA play a significant role in promoting mucosal tolerance and shaping the microbiome (94).

**Figure 2:**
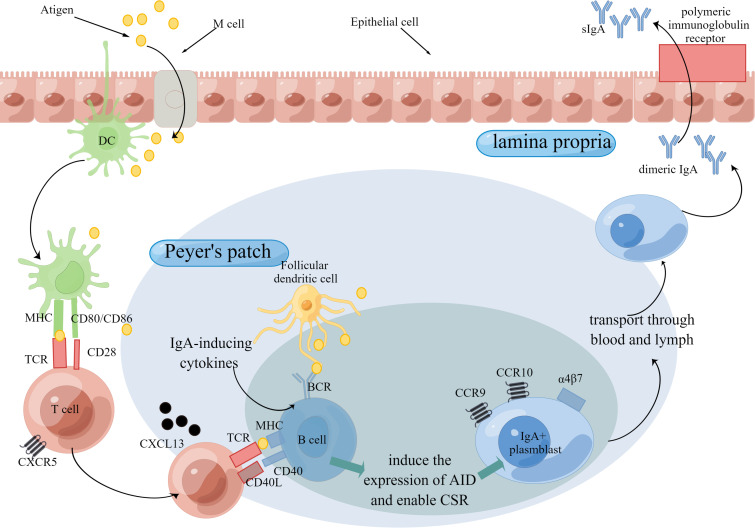
Production of IgA Through T cell-dependent Pathway. The intestinal mucosal immune function is mainly organized by the induction sites and effector sites. Peyer’s patch is the major induction site of immune responses. Antigens are delivered to Peyer’s patch by M cells and then may be captured and presented by DCs. In T cell-dependent pathway, B cells is stimulated by T cells, which release IgA-inducing cytokines, and then express CD40 ligand (CD40L) and cytokines that induce the expression of activation-induced cytidine deaminase (AID) and thereby enable class-switch recombination (CSR). After that, IgA^+^ plasmblast is transported to effector sites, such as lamina propria. Aging is detrimental to the production of IgA for the reason that aging adversely influences the number or/and function of immune cells as well as immune molecules. Abbreviations: TCR, T cell receptors; BCR, B cell receptors.

There are fewer studies on sIgM. An older study showed that sIgM secretion in the intestinal mucosa appears to not correlate with aging (95). Compared to specific sIgA secretion upon antigen stimulation, nonspecific sIgA secretion in intestinal mucosa seems to be a poor indicator of mucosal immunity function (77). Considering this, more researchers focus on specific sIgA secretion. A couple of studies showed reduced antigen-specific sIgA secretion in the intestinal mucosa (77, 96, 97), whose mechanism involves the immune cells and immune molecules during sIgA production (**Figure 2**). sIgA serves as the first line of defense in the intestinal mucosa, the reduced secretion of which will do harm to antigen-specific immune responses.

## 4 Interaction between gut microbiota and mucosal immunity during aging

### 4.1 The impact of microbiome in the aging gut on mucosal immunity

With aging, the age-related changes of the intestinal microbial composition, called microbial dysbiosis, are mainly characterized in the elderly. The abundance of core commensal bacteria decreases, while the abundance of opportunistic and pathogenic microorganisms increases (98). This change implies that the aging intestine loses protection from commensal bacteria and at the same time the growth and colonization of pathogens increase. It can impair the intestinal mucosal barrier by directly damaging the TJ or modulating signaling pathways related to the TJ (99), leading to leakage of pathogens, metabolites of pathogens, and pro-inflammatory cytokines. Further, it increases the risk of intestinal infections, triggers chronic intestinal inflammation, and exacerbates the process of aging (100, 101). There have been some researches on the underlying mechanisms of the impact of specific microorganisms. For example, Strains Lactobacillus reuteri 5454 can induce Tregs and Reg3b expression. Bifidobacterium animalis spp. lactis 5764 can promote the maturation of DCs and the secretion of IL-17A (102). And Bifidobacterium lactis Bi-07 can enhance phagocytic activity of monocytes and granulocytes (103). Also, Bifidobacterium lactis HN019 can enhance the function of natural killer cells and polymorphonuclear cells (104).

In addition, the intestinal microbiota can affect the intestinal mucosal immune function through its metabolism. With aging, the potential of saccharolytic decreases, the ability of the colon to utilize lactate decreases, the catabolism of creatine increases, and the production of cobalamin, biotin as well as short chain fatty acids (SCFAs) decreases. Also, the bile acid pool composition is affected (105, 106). These microbial metabolites play a key role in regulating immune responses. For instance, SCFAs can inhibit NF-kB, stimulate IL-8 secretion (107), induce Treg differentiation (108), enhance regulatory B cell differentiation, and inhibit differentiation of plasma cells (109). Consequently, SCFAs have an anti-inflammatory effect and the reduced SCFAs can increase the intestinal inflammation. Bile acids can induce monocyte differentiation to IL-12 hypo-producing DCs and inhibit the production of pro-inflammatory cytokines and chemokines in monocytes and macrophages (110, 111). Furthermore, high fecal lactate is associated with ulcerative colitis and other inflammatory bowel diseases (112). In conclusion, the intestinal microbiota, through changes in its composition and metabolism, can influence the immune function of the intestinal mucosa.

### 4.2 The impact of mucosal “immunosenescence” on intestinal microbiome

The mucosal immune system shapes the intestinal microbiome by allowing commensal bacteria to occupy mucosal ecological niches, while selectively eliminating or neutralizing harmful microorganisms. The mucosal “immunosenescence” can directly affect intestinal recognition and processing of gut microbiota (113, 114). Also, it promotes intestinal microbial dysbiosis by other mechanisms. For example, the responses of sIgA to Clostridiaceae and Enterobacteriaceae decrease with aging (115). Additionally, the increased intestinal inflammation in aging individuals can promote microbial dysbiosis. For instance, in the aging TNF-deficient mice, protected from age-associated inflammation, researchers did not observe age-related microbiome changes (116).

## 5 Strategies to slow down intestinal aging

“immunosenescence”, state of immune dysfunction, results in an increased susceptibility to infections, reduced abilities to heal, and altered homeostasis (2). As the largest immune compartment, intestinal mucosal immune function is strongly influenced by aging.

Aging impairs the intestinal mucosal barrier, which brings about the intestinal mucosal immune dysfunction. First, aging affects the number of ISCs, which is related to changes of crypt. Also, aging adversely influences the regeneration and self-renewal of intestinal epithelium. Second, aging affects the differentiation of ISCs. That is to say, aging affects the composition of IECs. Further, aging the function of IECs. Also, aging increases intestinal permeability by changing the expression of key TJ proteins, resulting in the impairment in the physical barrier. At last, Aging affects extracellular components of intestinal mucosal barrier, including the production and function of mucus and AMP. Understanding the impact of aging on the intestinal mucosal barrier will help explore strategies to slow down intestinal aging. Low caloric states can enhance ISC function during aging by inducing fatty acid oxidation (31). And calorie restriction can inhibit mTORC1 to promote epithelial regeneration (117). Additionally, Wnt supplementation, Notum inhibitors, and inhibition of Cdc42 activity can restore ISC function as well (**Figure 1**). Blocking pro-inflammatory cytokines associated with increased permeability is one of the strategies. For instance, Nicotinamide mononucleotide supplementation can downregulate the TNF-α expression and upregulates the ocludin as well as claudin-1 expression (118).

Aging can affect the intestinal mucosal innate and adaptive immune function through immune cells and immune molecules. On the one hand, aging affects intestinal mucosal immune function through immune cells. First, aging affects the ability of DCs to induce a balance of regulatory and effector T cell response through the impaired response to Retinoic Acid, the reduced cytokine secretion, and the costimulatory signal expression. It is detrimental to antigen-specific immunity and the tolerance of intestinal harmless antigen. In addition, aging diminishes the immune function of T cells. Aging affects the proliferative response of PP CD4^+^ T cells, which is disadvantageous to antigen-specific immunity. Aging affects the expression of immunomodulatory molecules in LP CD4^+^ T cells, which may be associated with CD4^+^ T cells hyperactivation as well as mucosal inflammatory environment. Aging affects the frequency and function of Th17 cell, which is disadvantageous to antigen-specific immunity and mucosal barrier integrity. Understanding the impact of aging on immune cells can help explore strategies targeting immune cells to slow down intestinal aging. Here are some examples. Exogenous addition of IL-15 can enhance the antigen-presenting ability of DCs by upregulating the costimulatory molecules CD80 and CD86, which introduce effector T cells to recognize antigens and promote pathogen-specific immune responses (59). Increasing the response of CD4^+^ T-cell to cytokines, such as adipose-derived mesenchymal stem cell transplantation, can improve the response of CD4+ T cells to cytokine and increase the antigen-specific sIgA (119). And Exogenous addition of cytokines synthesized by aging CD4^+^ T cells, such as IL-4, IL-5, and IL-2, may improve immune function (120, 121). In addition, it is found that CTLA-4 and PD-1 blockers widely used for cancer treatment could lead to unintended immune-mediated intestinal inflammation (122). And anti-CTLA-4 treatment confers the anti-inflammatory property to the colitis model mouse by inducing IL-10-producing Tregs (123). However, there is a lack of studies on intestinal aging. So the effect of CTLA-4 and PD-1 blockers to intestinal aging remains unknown. On the other hand, aging affects intestinal mucosal immune function through immune molecules. Aging remodels cytokine expression patterns, tending to a pro-inflammatory phenotype of the intestinal and systemic immune system. These cytokines play important roles in nearly all aspects of immunity. So, the age-related changes of cytokines will cause adverse effects on intestinal mucosal immune function. Recombinant cytokines and cytokine blockers are potential strategies to slow down intestinal aging. Although studies on aging are lacking, the widespread use of cytokines in IBD suggests that they are very promising strategies (124). Additionally, aging affects the secretion of antigen-specific IgA in the intestine, which does harm to intestinal adaptive immunity. Targeting cells and chemokines, involved with the production and transport processes of antigen-specific sIgA, can promote pathogen-specific immune function.

At last, the microbiome in the aging gut and the aging mucosal immune system are closely related. The altered composition and metabolism of the gut microbiome adversely affect the intestinal mucosal immunity. Conversely, the aging mucosal immune system directly and also indirectly affects the composition of the gut microbiota. Therefore, modulation of gut microbiota is an important strategy to slow down intestinal aging, including dietary interventions, exercise interventions, drugs and bacterial therapies (125).

## Author contributions

SZ devised the study. HZ, CZ, and QW were involved in the conception of the study and critically revised the manuscript. SF and YF were involved in writing the article. All authors agree to be accountable for the content of the work.

## Funding

This work was supported by the National Natural Science Foundation of China (82074214). Funding was also provided by the Research Fund Project of Zhejiang Chinese Medical University (2019ZY02).

## Conflict of interest

The authors declare that the research was conducted in the absence of any commercial or financial relationships that could be construed as a potential conflict of interest.

## Publisher’s note

All claims expressed in this article are solely those of the authors and do not necessarily represent those of their affiliated organizations, or those of the publisher, the editors and the reviewers. Any product that may be evaluated in this article, or claim that may be made by its manufacturer, is not guaranteed or endorsed by the publisher.
